# Imaging Features of Pulmonary Infections

**DOI:** 10.7759/cureus.116685

**Published:** 2026-09-21

**Authors:** Alan Campbell, Sarim Ather, Christopher Conlon, Zeshan Riaz, Gillian Kiely, Aman Gupta, Fergus Gleeson

**Affiliations:** 1 Radiology, St George's University Hospitals NHS Foundation Trust, London, GBR; 2 Radiology, Oxford University Hospitals NHS Foundation Trust, Oxford, GBR; 3 Nuffield Department of Medicine, Oxford University Hospitals NHS Foundation Trust, Oxford, GBR; 4 Medical Affairs, Pfizer Ltd, Sandwich, GBR

**Keywords:** bacterial, ct, fungal, infection, mycobacterial, pneumonia, pulmonary, radiograph, thoracic radiology, viral

## Abstract

Pulmonary infections are common in hospitals. Radiological imaging is crucial for rapid diagnosis, revealing infection patterns and distinguishing them from non-infectious conditions. This review highlights key radiological features that aid in diagnosing pulmonary infections and identifying causative organisms to guide therapy.

Bacterial pneumonias may show lobar, bronchial, or interstitial patterns, characterised by lobar consolidation, bronchial wall thickening, and alveolar septal thickening, respectively. Viral infections typically present a multifocal ground-glass pattern. An airway-centric pattern, with “tree-in-bud” opacification and bronchial wall thickening, may also occur, often coexisting with bacterial infections, which can complicate diagnosis. Fungal infections, particularly dangerous for immunocompromised individuals, can be suggested by CT imaging features such as the halo, reverse halo, and air-crescent signs.

Given the complexities and limitations of timely diagnosing pulmonary infections, accurately interpreting radiological findings is vital. Enhancing our ability to use imaging effectively enables healthcare professionals to make better prescribing decisions, aiming for more targeted therapies and improved patient outcomes.

## Introduction and background

Lower respiratory infections remain the deadliest communicable disease group and were the fifth leading cause of death worldwide in 2021 [1]. Although they affect people of all ages, they cause disproportionate morbidity and mortality in the elderly and immunocompromised, whose numbers are growing [2].

Complex patients presenting with difficult-to-treat underlying pathogens create a diagnostic challenge that necessitates precise and timely identification to avoid incorrect or delayed treatment. Fungal infections, in particular, pose a significant challenge due to their rapid progression and high fatality rate, especially in immunocompromised individuals. Similarly, viral infections can present with multifocal patterns that complicate differentiation from bacterial infections, with both types often present concurrently, further clouding the diagnostic picture [3].

Although empirical treatment targeting the most likely organism is often started immediately, the risks associated with broad-spectrum therapies (e.g., antimicrobial resistance) and the high mortality associated with misdiagnosis warrant pursuing a specific diagnosis. Thus, the importance of detailed imaging cannot be overstated [4].

Early imaging with chest radiographs and computed tomography (CT) is an important component of diagnostic evaluation. It is more readily accessible than invasive procedures such as biopsies and bronchoalveolar lavage and provides more rapid diagnosis compared with culture samples. Imaging thus complements these and other diagnostic techniques. Effective imaging can help distinguish between different types of infection and suggest the likely causative agent, helping to inform appropriate and effective treatment. However, as with all diagnostic tools, radiological tests do have limits and do not have perfect specificity or sensitivity in lung infection diagnosis [5].

This narrative, pictorial review is based on a non-systematic search of PubMed and Google Scholar (using terms such as “pulmonary infection,” “pneumonia,” “CT,” “chest radiograph,” and organism-specific terms) together with standard radiology reference texts. Representative and illustrative sources were selected at the authors' discretion, and no formal systematic search, risk-of-bias assessment, or quantitative synthesis was undertaken. All figures are original clinical images from the authors' own archives at Oxford University Hospitals NHS Foundation Trust, fully anonymised, and are reproduced with appropriate local approval; no images are reproduced from third-party sources, and no reproduction permissions were, therefore, required.

Table 1 details key terms and signs referred to within this review.

**Table 1:** Key imaging terms and signs used throughout this review.

Term / sign	Meaning or appearance	Typical significance
Ground-glass opacity (GGO)	Hazy increased attenuation that does not obscure underlying vessels or bronchi	Partial alveolar filling; non-specific (viral, atypical, fungal, early infection)
Consolidation	Homogeneous opacification obscuring vessels, often with air bronchograms	Alveolar filling; typical of lobar bacterial pneumonia
Centrilobular nodules	Small nodules centred on the core of the secondary pulmonary lobule	Airway-centred (bronchiolar) disease
Tree-in-bud	Branching centrilobular nodular opacities resembling a budding tree	Small-airway infection/inflammation (TB, viral, aspiration)
Halo sign	Nodule or mass surrounded by a rim of GGO	Haemorrhage around an angioinvasive lesion; early angioinvasive aspergillosis
Reversed halo (atoll) sign	Central GGO encircled by a ring of consolidation/density	Seen in mucormycosis (also organising pneumonia, infarct)
Air-crescent sign	Crescent of air between a cavity wall and an intracavitary mass	Retracting necrotic tissue or fungal ball; recovery phase of angioinvasive aspergillosis, or aspergilloma
Crazy paving	GGO with superimposed inter- and intralobular septal thickening	Advanced alveolar disease (COVID-19, PCP)
Cavitation	Gas-containing space, possibly within consolidation or a nodule	Necrosis; TB, *S. aureus*, *Klebsiella*, fungi

## Review

Bacterial infections

Bacterial pneumonia is common and results from bacterial colonisation and a buildup of inflammatory products within the lung. Bacterial lung infection can demonstrate a number of patterns on imaging (as respectively demonstrated in Figure 1) [5].

**Figure 1:**
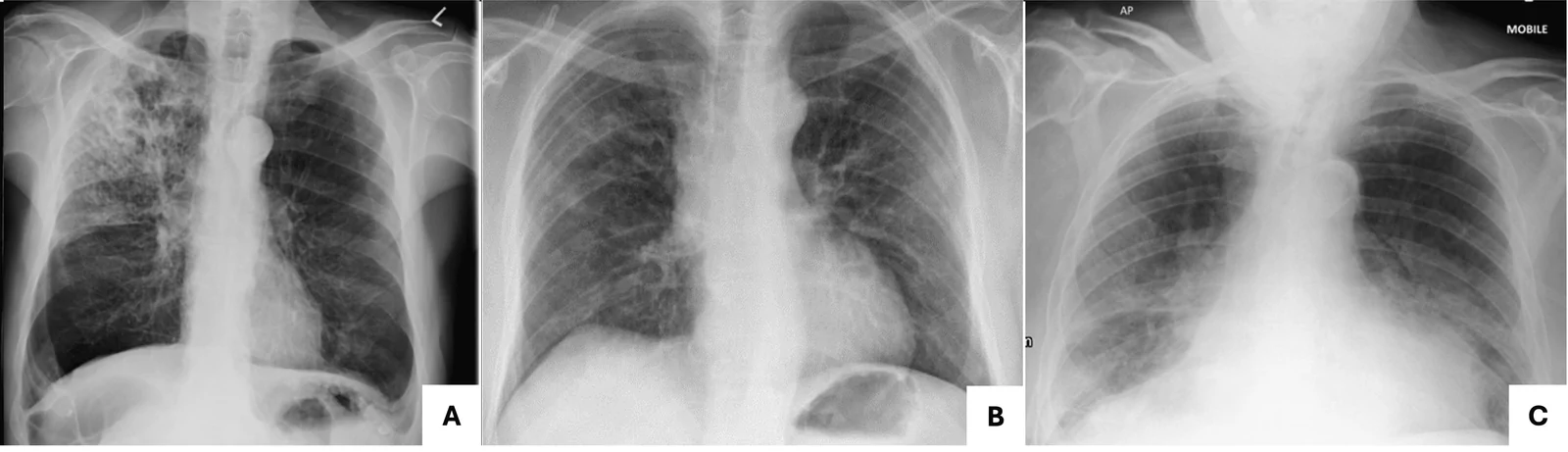
Example radiographs demonstrating A) lobar pneumonia, B) bronchopneumonia and C) interstitial pneumonia.

Lobar Pneumonia

Inflammatory exudates collect within the alveoli, and the infection spreads to adjacent segments through interalveolar pores of Kohn, leading to confluent areas of consolidation, confined to a single segment or lobe. On CT, there might be adjacent ground-glass opacities (GGOs) caused by partial alveolar filling. Infection can spread between lobes via incomplete major fissures [5].

Bronchopneumonia

The infection leads to bronchial wall thickening, mucus plugging, and peribronchiolar inflammation. This appears as centrilobular nodules, tree-in-bud pattern, and peribronchiolar consolidation with a patchy non-segmental distribution [5].

Interstitial Pneumonia

Inflammatory cells infiltrate the alveolar septa and interstitial tissues, leading to septal thickening, nodules, and areas of GGO on imaging [5].

As many pathogens can be responsible for pneumonia with more than one pattern, pneumonias are categorised as community-acquired, hospital-acquired, or aspiration pneumonia, as this helps narrow down the likely causative organisms and guide empirical therapy [6].

Community-Acquired Pneumonia

As the name suggests, community-acquired pneumonia (CAP) refers to infections acquired outside of healthcare facilities, by definition (or becoming apparent within the first 48 hours of admission, beyond which it is termed hospital-acquired pneumonia (HAP)). Designation of infection as CAP is based upon this “pre-hospital acquisition” definition and is not specific to a particular pathogen; similarly, the pathogens commonly involved in CAP are not specific to it. *Streptococcus pneumoniae*,* Haemophilus influenzae*,* Mycoplasma pneumoniae*,* Chlamydia pneumoniae*, and *Legionella pneumophila *are the most common causative organisms in CAP [6, 7].

*Streptococcus pneumoniae *has been identified in 33-50% of predominantly hospitalised adults in developed countries with community-acquired pneumonia in whom a microbial aetiology could be established [8]. It usually demonstrates a lobar pattern with a preference for the lower lobes, but multi-lobe involvement is also common [9]. Some evidence suggests parapneumonic effusions are becoming more common in the UK, associated with serotypes 1 and 3 despite vaccination programmes [10]. Mild lymph node enlargement may be evident [11].

*Haemophilus influenzae *pneumonia can occur in children and older patients, and particularly in smokers and those with chronic lung disease or immunocompromised status, and is seen in both CAP and HAP (discussed later in the article). The nasopharynx is often colonised by this organism, which can cause pneumonia when translocated to the lower respiratory tract [12]. There is usually a peripheral predominance, with GGO, centrilobular nodules and consolidation all being common radiological features [13].

Staphylococcal pneumonia is a relatively rare cause of CAP, implicated in around 5% of cases, but is commonly the causative organism in HAPs and can also complicate viral pneumonias. The prevalence of *Staphylococcus aureus*, for example, in HAP has been reported as over 18% in Italian populations, and is acquired through haematogenous spread or aspiration. Imaging features include pulmonary consolidation, abscess, and cavitation and can have associated complex effusions [14, 15].

Pulmonary tuberculosis (TB)* *is caused by *Mycobacterium tuberculosis* and imaging features reflect different stages of infection (Figure 2) [16].

**Figure 2:**
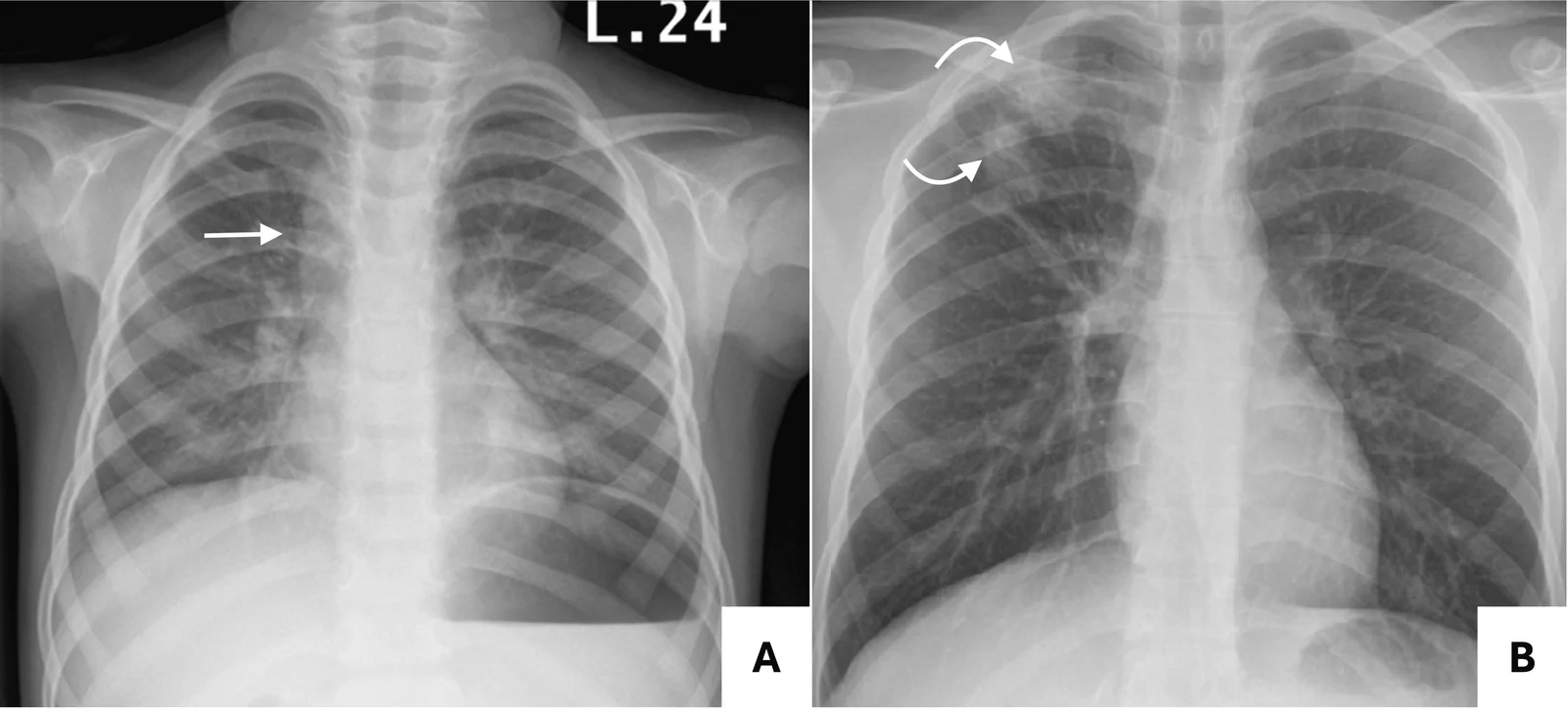
Radiographic patterns of pulmonary TB: A) Right unilateral mediastinal lymph node enlargement (straight arrow). B) Right apical pulmonary infiltrates (curved arrows).

Primary TB, where a patient is infected for the first time, manifests as (1) dense consolidation anywhere in the lung, particularly the mid- and lower zones in adults; (2) lymphadenopathy, particularly in children and typically right unilateral, and is often a differentiator when there are otherwise non-specific appearances of bacterial pneumonia; (3) miliary disease, which is rarer and usually seen in immunocompromised, infant, or elderly patients and where tiny randomly distributed nodules are present throughout the lungs; (4) pleural effusion, which is usually a delayed manifestation and essentially never seen in infants [16].

Post-primary TB occurs in patients who have had previous exposure and experience either re-infection or re-activation of infection; features are similar to those of primary TB but with the classic upper zone predilection, often with bilaterality, generally a lack of lymph node enlargement (although previously calcified nodes may be evident) and cavitation from exuberant immune response. Endobronchial spread is seen as tree-in-bud opacities on CT. Mucoid impaction can cause lobar collapse, as can bronchial stenosis, a fibrotic sequela of infection. Fibrotic scarring also causes parenchymal scarring, particularly at the apices, and traction bronchiectasis [16].

Non-tuberculous mycobacterial pulmonary infection is a separate entity, presenting as bronchiectasis and bronchial wall thickening and, on CT, GGO and tree-in-bud opacities, which can also cause suspicion of TB [16].

*Mycoplasma* spp.* *pneumonia usually affects young people. It is clinically characterised by dry cough, fever, and general fatigue. It can have a variable radiological appearance but most commonly demonstrates a bronchopneumonia pattern with bronchial wall thickening, centrilobular nodules and peribronchial consolidation. However, more widespread consolidation can also be seen. The radiographic appearance of *Chlamydia *spp.* *pneumonia is similar to that of *Mycoplasma *spp.* *and the two infections sometimes coexist [17].

*Legionella *spp. pneumonia is acquired by the inhalation of droplets of contaminated water, most frequently from water systems which become colonised by this pathogen [18]. Patients present with cough, dyspnoea, diarrhoea, headache, and myalgia. On CT, sharply marginated peribronchial consolidations within GGO are characteristic of *Legionella *spp. pneumonia [19].

Pulmonary nocardiosis predominantly affects immunocompromised patients. *Nocardia asteroides* is the most commonly implicated pathogen. Imaging demonstrates multifocal consolidation with abscess formation (Figure 3C). Cavitation develops in up to a third of cases, and centrilobular nodularity and peripheral GGO may be evident. In chronic infection, bronchiectasis, pleural thickening, and collections may develop [20]. 

**Figure 3:**
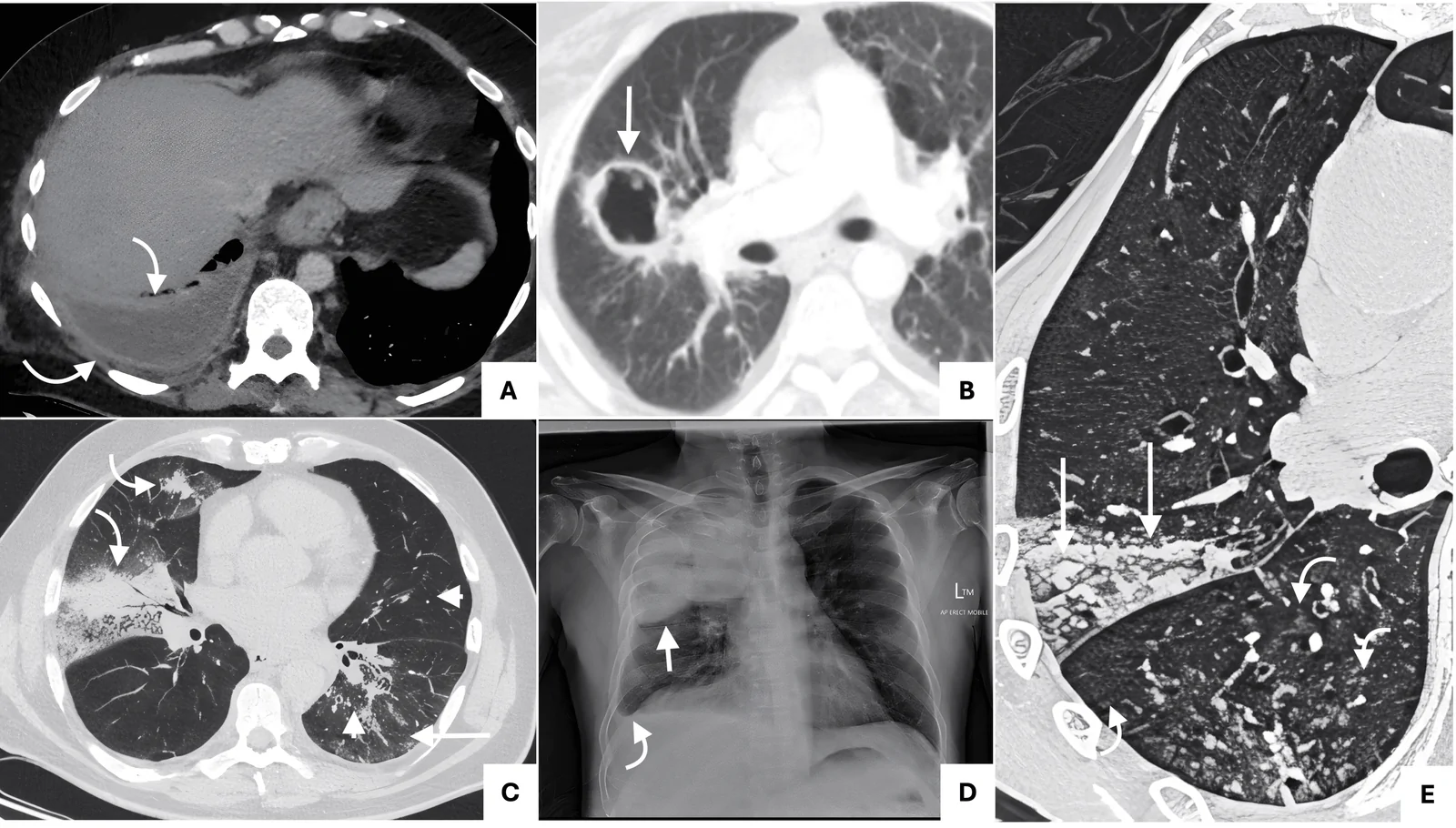
(A) A soft-tissue window CT of a right basal empyema demonstrating the split pleura sign, with enhancement and visualisation of the pleura (curved arrows). (B) Cavitation of the right upper lobe, demonstrated on CT (straight arrow). (C) Nocardiosis, demonstrated by multifocal areas of consolidation (curved arrows), centrilobular nodules (arrowheads) and peripheral ground glass opacities (straight arrow). (D) Lobar enlargement (straight arrow) and pleural effusion (curved arrow) in Klebsiella pneumonia. (E) Ground glass nodules (curved arrows) and plugging of the bronchi (straight arrows) in aspiration pneumonia.

Melioidosis, caused by* Burkholderia pseudomallei*, is a disease of the tropics, being especially endemic in northern Thailand and Queensland, Australia. Pulmonary infection is most common, usually manifesting as upper lobe infiltrates, with or without thin-walled cavitation, although diffuse infiltrates may be seen acutely. Lymph node enlargement is rare, which can help differentiate from post-primary TB [21].

Hospital-Acquired Pneumonia

Nosocomial or HAP is defined as a pneumonia occurring beyond 48 hours after admission and up to 48 hours after discharge from hospital. The infection is often caused by multiple organisms, including Gram-negative bacilli (Enterobacterales, including* E. coli,* and the aerobic non-fermenting *Pseudomonas aeruginosa*), and Gram-positive cocci (*S. aureus* and *S. pneumoniae*) [22].

The infections are more frequent and severe in intensive care (ICU) patients. Mechanical ventilation is a major risk factor since it can facilitate bacterial dissemination, and sputum clearance is reduced due to an impaired cough. However, HAP can also occur in non-ventilated ICU patients [22].

The radiographic pattern of HAP is variable. Most commonly, patients demonstrate unilateral or bilateral consolidation, but nodules and GGO may also be seen. Differentiating HAP from other pulmonary abnormalities frequently seen in ICU patients such as pulmonary infarction, oedema or acute respiratory distress syndrome (ARDS) can be challenging [23].

Pulmonary infiltrates due to both pulmonary oedema and ARDS tend to be bilateral, symmetrical and with an anterior-to-posterior gradient. Focal asymmetrical consolidation favours infection over oedema [23].

Aspiration Pneumonia

Inhalation of oropharyngeal or gastric contents into the respiratory tract can lead to aspiration pneumonia. The aspirated contents may consist of bacterial flora, gastric acid or food debris, which can respectively lead to bacterial pneumonia (usually anaerobic), chemical pneumonitis or granulomatous reaction. Imaging findings include airway plugging with aspirated contents and associated atelectasis, centrilobular nodules with surrounding GGO, and bilateral consolidation in the dependent areas of the lungs (Figure 3E) [24]. 

Complications

Immunocompromised patients are more susceptible to complications from bacterial pneumonia, which can occur with both CAP and HAP infections. Parapneumonic pleural effusions (Figure 3A) may develop in up to 60% of hospitalised pneumonia patients. While in most cases these are reactive and resolve with antibiotic therapy, in around 5% of cases, they become complicated and progress to empyema [25].

Lobar enlargement occurs with an extensive exudative process. While the finding was initially seen as specific for *Klebsiella *spp. pneumonia, other suppurative processes, including tuberculosis, can also cause lobar enlargement (Figure 3D) [26]. 

Cavitation can occur in immunocompetent patients, for example with *Streptococcus pneumoniae, Klebsiella pneumoniae* and *Staphylococcus aureus*; right upper lobe cavitation is shown in Figure 3B. Cavitation can also be seen in *Aspergillus *spp.* *infections in immunocompromised patients [26]. As discussed above, cavitation is also a feature of post-primary TB [16, 26].

Table 2 summarises the imaging appearances of bacterial pulmonary infection.

**Table 2:** Summary of the imaging appearances of bacterial pulmonary infection. Sources: References [8-24, 26].

Organism / entity	Typical imaging pattern(s)	Key differentiating clue(s)
Streptococcus pneumoniae	Lobar consolidation, lower-lobe preference; multilobar common; ± adjacent ground-glass opacity (GGO)	Commonest community-acquired pneumonia (CAP); parapneumonic effusion common, lymphadenopathy uncommon
Haemophilus influenzae	GGO, centrilobular nodules and consolidation, peripheral predominance	CAP and hospital-acquired pneumonia (HAP); risk in COPD, smokers, chronic lung disease
Staphylococcus aureus	Consolidation, abscess, cavitation, complex effusions	Common in HAP; complicates viral pneumonia; haematogenous or aspiration spread
Mycoplasma pneumoniae	Bronchopneumonia: bronchial wall thickening, centrilobular nodules, peribronchial consolidation; may be widespread	Young patients; dry cough, fever, fatigue
Chlamydia pneumoniae	Similar to Mycoplasma	May coexist with Mycoplasma
Legionella pneumophila	Sharply marginated peribronchial consolidation within GGO	Waterborne; diarrhoea, headache, myalgia
*M. tuberculosis *(primary)	Dense consolidation (mid/lower zones), lymphadenopathy, miliary nodules, effusion	Lymphadenopathy (esp. children, right unilateral) is a key clue
*M. tuberculosis* (post-primary)	Upper-zone cavitation, tree-in-bud, fibrosis, traction bronchiectasis	Upper-zone predilection; usually no lymphadenopathy
Non-tuberculous mycobacteria	Bronchiectasis, bronchial wall thickening, GGO, tree-in-bud	Mimics TB
Nocardia asteroides	Multifocal consolidation with abscess, cavitation (up to 1/3), centrilobular nodules, peripheral GGO	Immunocompromised
Klebsiella pneumoniae	Lobar consolidation with lobar (bulging-fissure) enlargement, cavitation	Lobar enlargement a classic clue (not specific)
*Burkholderia pseudomallei* (melioidosis)	Upper-lobe infiltrates ± thin-walled cavitation; diffuse infiltrates acutely	Tropics (N Thailand, N Australia); rare lymphadenopathy distinguishes from post-primary TB
Hospital-acquired pneumonia (mixed: Gram-negative bacilli, *Pseudomonas*,* E. coli*; *S. aureus*, *S. pneumoniae*)	Variable: uni/bilateral consolidation, nodules, GGO	Onset >48 h after admission; focal asymmetrical consolidation favours infection over oedema/acute respiratory distress syndrome
Aspiration pneumonia	Airway plugging with atelectasis, centrilobular nodules with surrounding GGO, dependent consolidation	Dependent (gravity-related) distribution

Viral infections

There is significant overlap in the imaging appearances of viral infections, and it is thus not possible to make a specific aetiological diagnosis of a viral pneumonia on imaging alone. However, viral infections are a frequent occurrence in immunocompromised patients, with cytomegalovirus, herpes simplex virus, measles and adenovirus being common causative organisms [27].

Imaging patterns can be variable and affected by the immune status of the host, with more progressive and diffuse imaging abnormalities being more common in immunocompromised patients. It is thus important to consider history and examination findings, with insidious onset, disproportionately high tachycardia and tachypnoea compared to pyrexia being indicators of viral infection over bacterial. Moreover, co-infection with bacteria is common. Nonetheless, recognising a viral pneumonia can prevent the presumption of bacterial infection and allow for more judicious use of antibiotics [27].

Viral infections predominantly demonstrate a multifocal GGO pattern and tend to be more diffusely distributed than bacterial infections. Airway-centric abnormalities may be encountered, with tree-in-bud opacification and bronchial wall thickening. Cavitation, lymphadenopathy, or pleural effusions are not common findings [28]. We will now outline the typical imaging features of common and important viral pneumonias.

Most influenza viruses often cause mild to moderate seasonal upper respiratory tract infections in immunocompetent patients. In immunocompromised patients, those with chronic diseases, the elderly, or infants, severe complications such as haemorrhagic bronchitis or pneumonia may occur. In early stages of the disease, tracheobronchitis or neutrophilic bronchopneumonia patterns may be demonstrated. In later stages, this progresses to diffuse alveolar damage with intra-alveolar oedema and haemorrhage [29].

On imaging, this manifests as bilateral patchy GGO/consolidation and ill-defined small nodules, particularly in the lower lobes (Figure 4A). Pleural effusions are rare. Development of lobar consolidation indicates secondary bacterial pneumonia [30].

**Figure 4:**
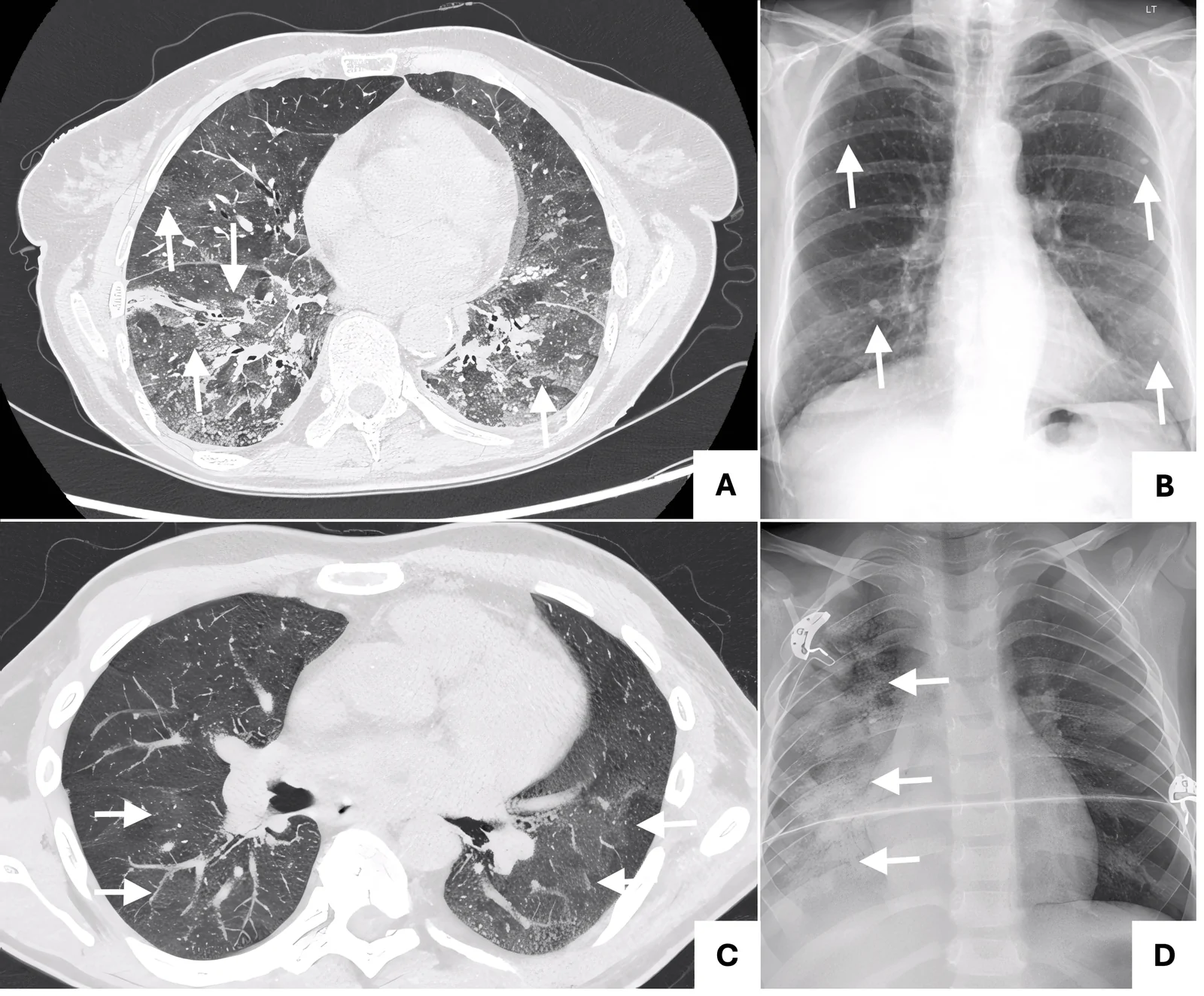
(A) CT image of Influenza viral pneumonia, with patchy ground glass opacities (e.g., straight arrows). (B) Radiograph demonstrating the small calcified pulmonary nodules of a recovered Varicella-zoster virus infection (straight arrows). (C) CT image demonstrating segmental ground glass opacification in pulmonary Herpes simplex virus infection (straight arrows). (D) Patchy right-sided opacification in Adenovirus infection (straight arrows).

Human parainfluenza virus (HPIV) infection demonstrates a variety of clinical manifestations, which include conjunctivitis, otitis media, croup, pharyngitis, and pneumonia. It is the second most common virus in patients receiving care in intensive treatment units and is a common cause of death in patients with haematological malignancies [31]. CT shows multiple areas of patchy GGO and consolidation and bronchial wall thickening. Centrilobular nodules may be present. The features are difficult to differentiate from bacterial pneumonia, and bacterial co-infection is a common occurrence [32].

Adenovirus accounts for approximately 5-10% of paediatric respiratory tract infections [33, 34]. Immunocompetent patients present with mild upper respiratory symptoms which resolve within two weeks. In immunocompromised patients, the infection is more severe and can be a fatal condition [34].

On imaging, adenovirus pneumonia may resemble bacterial pneumonia with multifocal GGO and patchy consolidations in a segmental or lobar distribution. Changes may be unilateral or bilateral. Hyperinflation and atelectasis are frequently seen in infant and child populations. Long-term sequelae of adenovirus pneumonia include bronchiectasis, bronchiolitis obliterans, and unilateral hyperlucent lung (Swyer-James-Macleod) syndrome (Figure 4D) [28].

Herpes simplex virus (HSV) infection is predominantly seen in immunocompromised patients and can lead to necrotising tracheobronchitis, interstitial pneumonitis, or necrotising pneumonia. Patients with pulmonary infection may be pyrexial and have a productive cough, shortness of breath, and upper airway obstruction due to the formation of pseudomembranes resulting from tracheal ulcers [28].

On imaging, HSV pneumonia predominantly demonstrates multifocal segmental or subsegmental GGO with focal areas of consolidation (Figure 4C). Pleural effusion is common [35]. 

Varicella-zoster virus (VZV) pneumonia is a serious complication of disseminated VZV infection, which most commonly affects immunocompromised or pregnant patients, but can occur in previously healthy individuals. Clinical findings of a cutaneous rash and a history of contact with a chickenpox patient provide clues to diagnosis [36]. Chest radiographs demonstrate multiple small ill-defined nodules that may be confluent, as shown in Figure 4B. On CT, these nodules have a surrounding ground-glass halo (halo sign). Pleural effusions and hilar lymph node enlargement are rarer findings. Although the lung nodules may resolve soon after resolution of the cutaneous lesions, they may remain for several weeks, and even after full recovery, small, calcified nodules can persist [28].

Cytomegalovirus (CMV) infection is usually asymptomatic or causes mild flu-like symptoms in immunocompetent patients. In the immunocompromised population, pulmonary infections can be life-threatening. CMV infection is a frequent early complication (30-100 days post-transplant) of post-transplantation immunosuppression for solid-organ and haematopoietic stem cell transplants. The virus's pathophysiology is affected by host factors. In transplant recipients, a T-cell-mediated antigen response to infection induces severe necrotising pneumonia. Imaging findings are of asymmetric consolidation/GGO, small ill-defined centrilobular nodules, and thickened interlobular septa (see Figure 5A). In AIDS patients who are unable to mount an immune response due to a more profound immunodeficiency, pathogenic effects of CMV lead to direct lung damage, which appears as mass-like consolidation on imaging [27].

**Figure 5:**
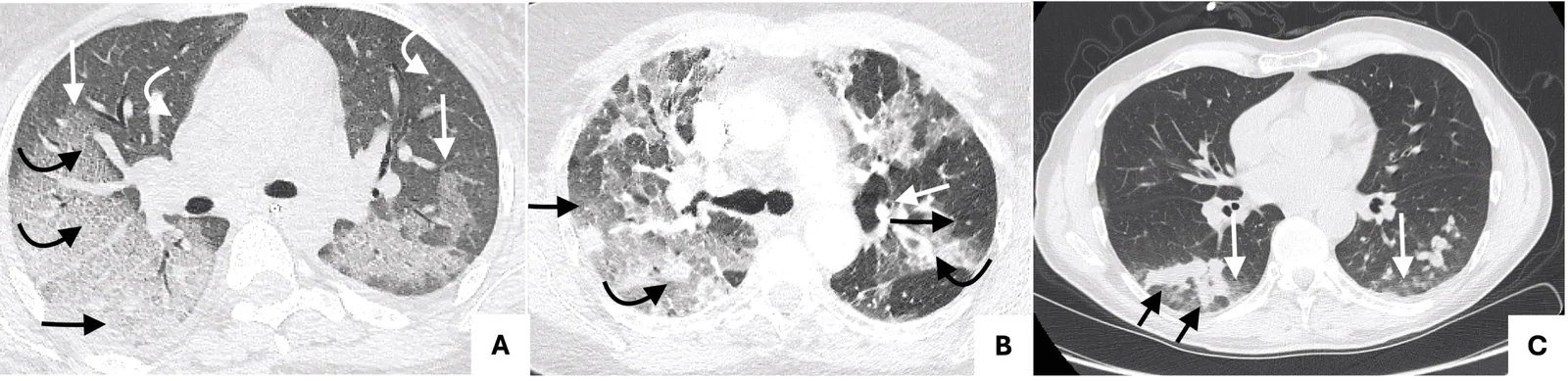
(A) Cytomegalovirus infection with bilateral, symmetric ground glass opacification (straight arrows) and consolidation (straight black arrow), poorly defined small centrilobular nodules (curved arrows), and thickened interlobular septa (curved black arrows). (B) Severe respiratory syncytial virus on CT, demonstrating some of the core features seen in this disease, including peribronchovascular consolidation (curved black arrows), bronchial wall thickening (straight arrow) and centrilobular nodules (straight black arrows). (C) CT image of human metapneumovirus, demonstrating bilateral branching opacities (straight black arrows), also with adjacent ground glass opacification (straight arrows).

Despite vaccination programmes, measles remains a cause of death in children and can lead to serious complications in pregnant women and immunocompromised patients [37]. On imaging, measles pneumonia shows peribronchial nodularity with thickened interlobular septa, which can progress to fibrosis. Pleural effusions and hilar lymph node enlargement are commonly seen [38].

Respiratory syncytial virus (RSV) infection can manifest as bronchiolitis, pneumonia, and asthma exacerbation in both children and adults. Immunocompromised patients and young children are at greater risk of severe pulmonary infection. CT shows bronchial wall thickening, centrilobular nodules, tree-in-bud opacity, and peribronchovascular consolidation (see Figure 5B) [28].

Human metapneumovirus (HMPV) is detected in 5.4% of respiratory infections in immunocompromised patients requiring hospitalisation [39]. It has a mortality of up to 40% in haematopoietic stem cell transplant recipients [40]. CT findings include bilateral ill-defined and branching centrilobular nodules and GGO (see Figure 5C). Pleural effusion is not common [41].

Coronaviruses 

Certain coronaviruses, such as severe acute respiratory syndrome coronavirus 2 (SARS-CoV-2), the virus that causes COVID-19, can cause life-threatening pneumonia in the immunocompromised patient. Typical findings on chest X-ray are multifocal bilateral airspace opacification with a peripheral and lower zone predominance (Figure 6A); similar features are seen in other viral pneumonias (such as severe acute respiratory syndrome (SARS)) [42] and acute eosinophilic pneumonitis. Pleural effusions are not uncommon in Middle East respiratory syndrome coronavirus (MERS-CoV) infection and can be seen in later, more advanced COVID-19, but they are rare in SARS. COVID-19 may present as unilateral patchy infiltrates in early disease, and tends to be peripherally distributed [43]. Overall, there is significant overlap in the appearances of these diseases. CT findings typically show bilateral GGO with or without consolidation, with a lower zone, peripheral and/or peribronchial predominance or a crazy paving appearance (GGO with superimposed intra- and interlobular septal thickening); see Figure 6B. The halo or atoll signs may be seen. Peripheral vessel enlargement within areas of airspace disease may also be demonstrated [43].

**Figure 6:**
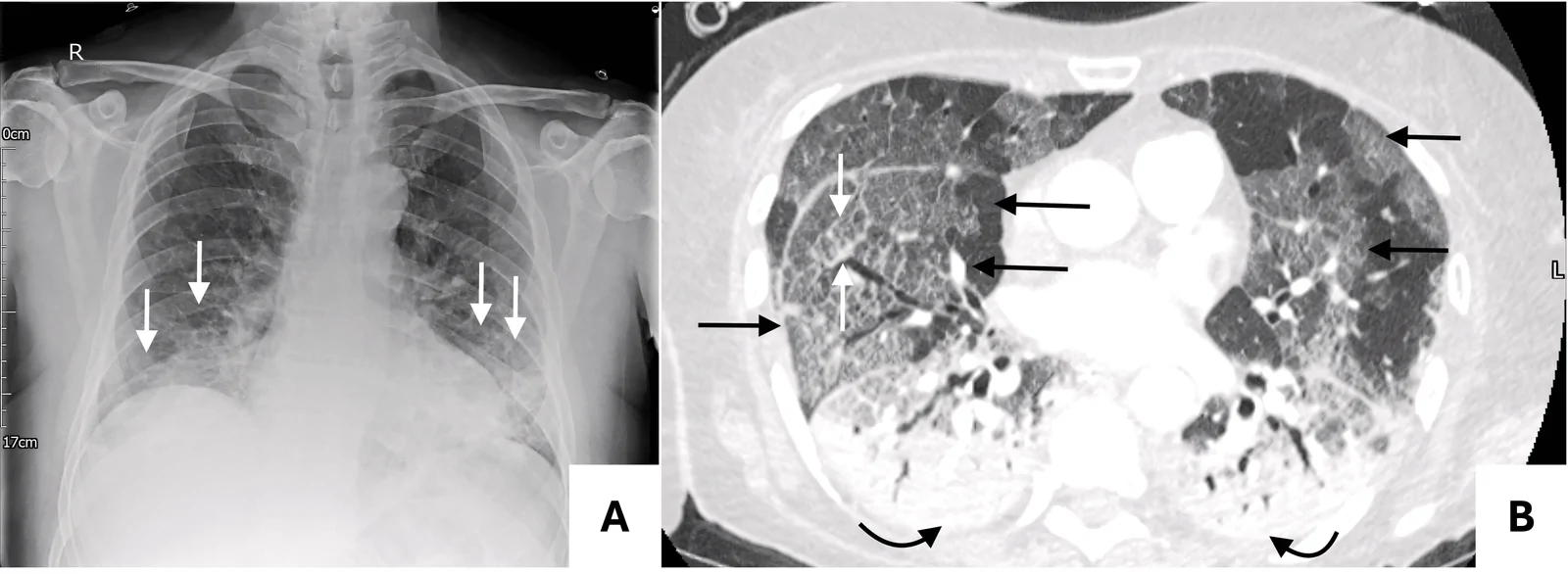
(A) Radiograph, with bilateral air space opacification (straight arrows). (B) CT image with bilateral ground glass opacification (straight black arrows), posterior consolidation (curved black arrows) and some crazy paving appearances (straight arrows), demonstrating the imaging features of COVID-19.

Table 3 summarises the imaging appearance of viral pulmonary infection.

**Table 3:** Summary of the imaging appearance of viral pulmonary infection. Sources: References [27-43].

Virus / entity	Typical imaging pattern(s)	Key differentiating clue(s)
General viral pattern	Multifocal GGO, diffusely distributed; ± tree-in-bud, bronchial wall thickening	Cavitation, lymphadenopathy and effusion uncommon; specific aetiology not possible on imaging alone
Influenza	Bilateral patchy GGO/consolidation, ill-defined small nodules, lower lobes	Effusion rare; new lobar consolidation suggests secondary bacterial infection
Parainfluenza (HPIV)	Patchy GGO and consolidation, bronchial wall thickening, centrilobular nodules	Hard to distinguish from bacterial; bacterial co-infection common
Adenovirus	Multifocal GGO, patchy segmental/lobar consolidation, uni/bilateral; hyperinflation and atelectasis in children	Sequelae: bronchiectasis, bronchiolitis obliterans, Swyer-James-Macleod
Herpes simplex virus (HSV)	Multifocal segmental/subsegmental GGO with focal consolidation; pleural effusion common	Immunocompromised; tracheal ulcers/pseudomembranes
Varicella-zoster virus (VZV)	Multiple small ill-defined nodules (may be confluent), halo on CT; later small calcified nodules	Cutaneous rash / chickenpox contact
Cytomegalovirus (CMV)	Asymmetric consolidation/GGO, small ill-defined centrilobular nodules, thickened interlobular septa	Transplant (30-100 days); mass-like consolidation in AIDS
Measles	Peribronchial nodularity, thickened interlobular septa, may progress to fibrosis	Effusions and hilar lymphadenopathy common
Respiratory syncytial virus (RSV)	Bronchial wall thickening, centrilobular nodules, tree-in-bud, peribronchovascular consolidation	Children and immunocompromised at greatest risk
Human metapneumovirus (HMPV)	Bilateral ill-defined branching centrilobular nodules and GGO	Effusion uncommon
Coronaviruses (SARS-CoV-2/COVID-19, SARS, MERS)	Bilateral GGO ± consolidation, peripheral/lower-zone/peribronchial; crazy paving; halo/atoll; vessel enlargement	Effusion rare in SARS, commoner in MERS and advanced COVID-19

Fungal infections

Pulmonary fungal infections demonstrate a wide variety of radiological manifestations including nodules, masses, consolidation, atelectasis, GGO, and cavities. Ancillary features, including lymphadenopathy and pleural effusions, may also be present [44, 45].

CT imaging detects lesions in early infection that would be missed with chest X-ray imaging [46]. The European Organisation for Research and Treatment of Cancer (EORTC) states that imaging features indicating a likely fungal aetiology of pulmonary infection include: the halo sign, reversed halo sign, hypodense sign, and air crescent sign [47].

Aspergillosis is a spectrum of diseases caused by the ubiquitous mould *Aspergillus*. Its spores are found both in indoor and outdoor air. *A. fumigatus* is the most frequently reported species in Europe and the US. *A. flavus* is increasingly being reported in rhino-orbito-cerebral infections in Asia [48]. Invasive pulmonary aspergillosis causes significant mortality and morbidity in the immunocompromised population [49].

Angioinvasive aspergillosis usually manifests within two weeks of the onset of immunosuppression, at which point neutropenia is usually most marked. Fungal hyphae invade the lung vasculature, precipitating thrombosis, pulmonary infarction, and alveolar haemorrhage. Characteristic imaging features (Figure 7A) include multifocal, poorly defined peripheral nodules that begin to join into more confluent masses. Lobar or diffuse pulmonary consolidation is common, but less specific. The nodules may demonstrate the halo sign, central necrosis, and cavitation. Retraction of the tissue within the cavity produces the air-crescent sign and can indicate a good prognosis. Pleural effusions and lymphadenopathy are uncommon findings, but chest wall or mediastinal invasion can occur [50].

**Figure 7:**
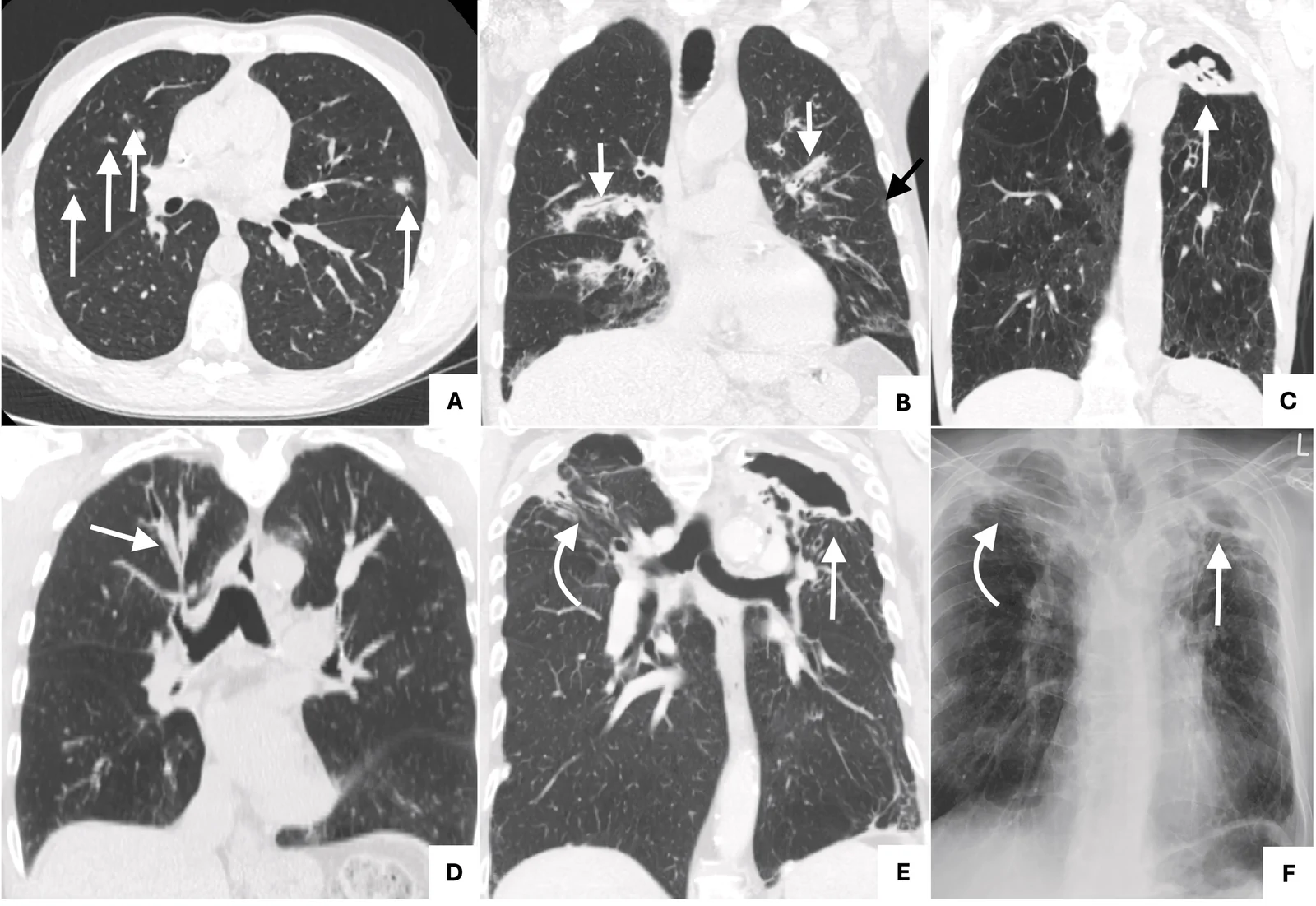
(A) CT image demonstrating the appearances of angioinvasive aspergillosis, of pulmonary nodules with the halo sign (straight arrows). (B) CT image demonstrating the features of airway invasive aspergillosis, with nodular airway thickening (straight arrows) and some peripheral tree-in-bud opacification (straight black arrow). (C) Coronal CT image demonstrating the air-crescent sign, with a fungal ball within a thick-walled left upper lobe cavity (straight arrow). (D) Allergic bronchopulmonary aspergillosis, with large, branching foci of bronchial plugging, particularly evident in the right upper zone (straight arrow). These foci were dense on mediastinal windows. (E) Coronal CT and (F) radiograph images demonstrating upper zone fibrosis (curved arrows) and cavitation (straight arrows) in chronic fibrosing pulmonary aspergillosis.

Airway invasive aspergillosis is a less common manifestation of pulmonary aspergillosis, which is caused by invasion of the fungal hyphae into the airways. It leads to airway obstruction and nodular airway thickening and may lead to fistulation into the oesophagus and arteries. CT demonstrates tree-in-bud opacification or, less commonly, a bronchopneumonia pattern with patchy peribronchial consolidation (Figure 7B) [51, 52].

Chronic pulmonary aspergillosis is a localised, less aggressive manifestation of aspergillosis infection in the lung. It most commonly affects individuals with less profound immunosuppression, such as in diabetes, major nutritional deficits, and chronic systemic corticosteroid use. It may also affect patients with pre-existing lung damage such as tuberculosis, sarcoidosis, and emphysema. It tends to be upper lobe predominant and presents with consolidation, pulmonary calcifications, and cavitation (Figure 7E-7F). This creates a diagnostic challenge between this disease and other pathogens such as mycobacteria, actinomycosis and histoplasmosis [53].

There are three distinct forms of chronic pulmonary aspergillosis which differ from each other in clinical presentation and radiological appearance: (1) Chronic cavitating pulmonary aspergillosis is characterised by multifocal cavities, with an upper-lobe predilection and possible intra-cavity fungal ball formation. (2) Chronic necrotising pulmonary aspergillosis, also referred to as semi-invasive pulmonary aspergillosis, demonstrates cavitating nodules, consolidation and peri-lesional infiltrates. (3) Chronic fibrosing pulmonary aspergillosis demonstrates areas of parenchymal fibrosis, traction bronchiectasis and pleural fibrosis in addition to consolidation and cavitation [53, 54].

Aspergilloma refers to colonisation of a pre-existing lung cavity by a fungal ball made up of a confluence of intertwined fungal hyphae, combined with mucus and cellular material. The infection occurs in immunocompetent patients, and there is no tissue invasion. Sarcoidosis and tuberculosis are the most common underlying causes for cavitation [54].

On imaging, aspergillomas appear as a cavity containing a ball-like mass of soft-tissue attenuation (Figure 7C). The air crescent sign is typically evident, wherein a rim of air between the fungal ball and the cavity wall forms a crescent-shaped hypodensity. A reasonably specific sign of aspergilloma is the formation of fungal filaments between the wall of the cavity and the ball of fungus. Unlike the air-crescent sign in invasive aspergillosis, the fungus ball demonstrates no radiological change over a long period of time [41, 54-57].

Allergic bronchopulmonary aspergillosis (ABPA) occurs due to an allergic response to* Aspergillus *spp.within the airways of asthmatic individuals and those with cystic fibrosis. The hypersensitivity leads to bronchiectasis and mucus plugging, which manifests as a characteristic ‘gloved finger’ appearance (Figure 7D). ABPA can lead to the development of highly attenuating mucus plugs filling a dilated bronchus; this is a specific sign of ABPA. It suggests more severe disease and a higher risk of recurrence [58].

*Candida* species are a very rare and, due to frequent incidental colonisation, debated source of fungal pneumonia in immunocompromised patients. *Candida* pneumonia is suspected to have a mortality rate of up to 40%, and a *Candida*-bacterial synergy has been proposed as a potentiating entity. The most common imaging manifestation is patchy bilateral nodules with halo sign, GGO, and consolidation with relative sparing of the lung apices. Cavitation, mass formation, miliary pattern, and pleural effusions are not typical features (Figure 8A). It is important to note that isolation of *Candida* from respiratory samples usually reflects colonisation; a confident diagnosis generally requires histopathology or sterile-site culture [59-61].

**Figure 8:**
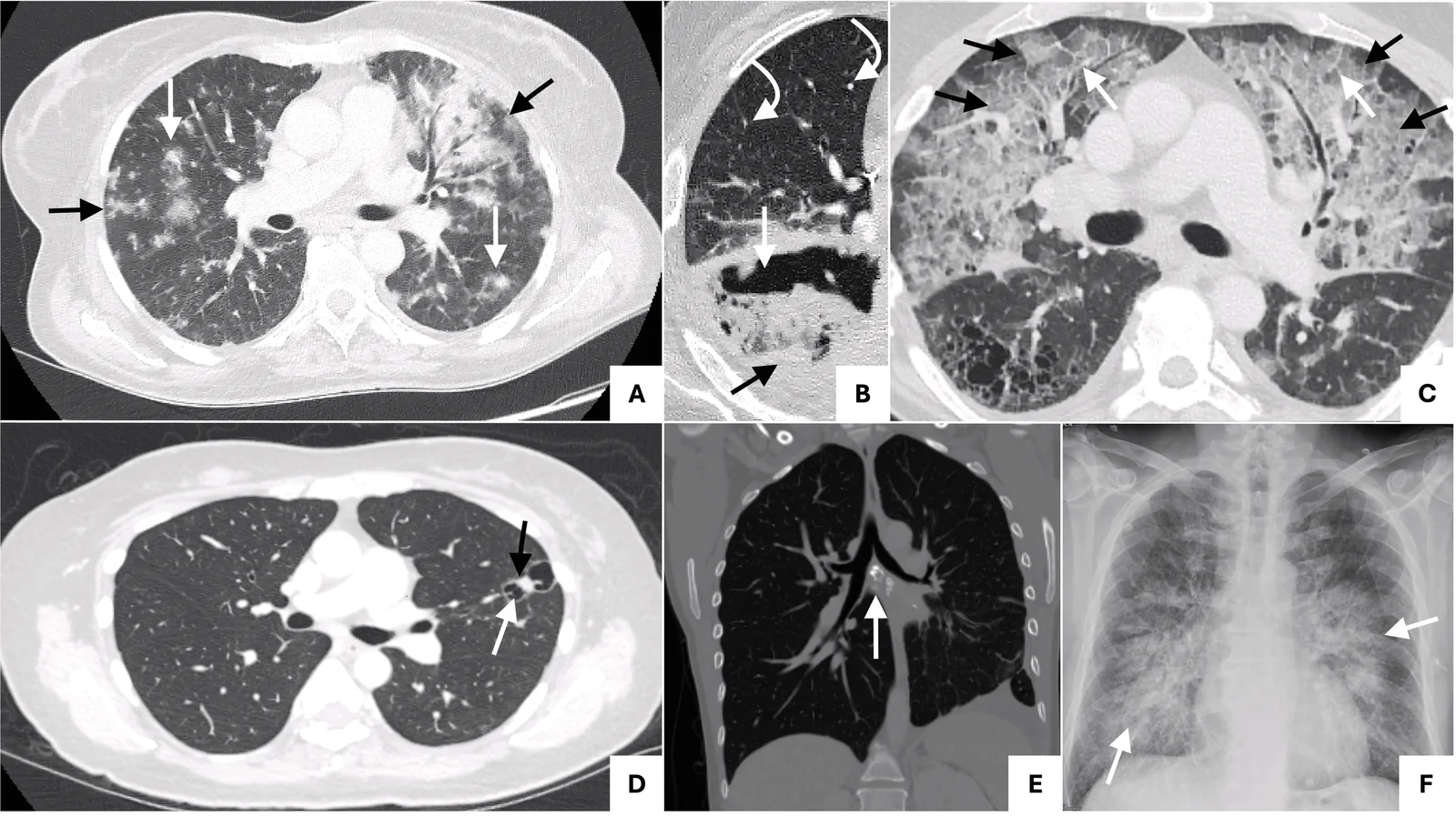
(A) Bilateral pulmonary nodules with surrounding ground glass haloes (straight arrows) and foci of ground glass opacification (straight black arrows) shown on CT in pulmonary candidiasis. (B) Mucormycosis on CT, with dense consolidation (straight black arrow) and cavitation (straight arrow), and with multiple pulmonary nodules (curved arrows). (C) Predominantly central ground glass opacification (straight black arrows) in Pneumocystis jirovecii pneumonia on CT; the crazy paving (straight arrows) appearance suggests advanced disease. (D) CT image of Cryptococcus, with peripheral cavitation (straight arrow) and nodules (straight black arrow). (E) Calcified lymph nodes (straight arrow) on coronal CT from previous Histoplasmosis infection. (F) Radiograph of Pneumocystis jirovecii pneumonia, with predominantly central diffuse opacification (straight arrows).

Infection of the small airway can result in airway wall thickening, dilatation, and obstruction, which manifests as a tree-in-bud pattern. Invasion of vessels can lead to pulmonary emboli due to obstruction of small and middle-sized arteries by *Candida* spores and pseudo-hyphae [59-61].

Mucormycosis infection is caused by inhalation of moulds of the order *Mucorales*. The reported mortality rate of pulmonary mucormycosis is wide, ranging from 29% (28-day mortality) to 80% (in-hospital mortality) [62-64]. The most distinguishing imaging feature is the presence of a reverse halo sign, which is significantly more common in mucormycosis, particularly in neutropenic patients, compared to other fungal infections. The clinical context and relatively rapid progression of imaging findings in mucormycosis can help differentiate the condition from other causes of reverse halo sign such as organising pneumonia, pulmonary infarcts, and lung cancer [65].

The remaining imaging findings are non-specific and include consolidation, solitary or multifocal nodules (possibly with the halo sign) and bronchial plugging. Vascular complications include perivascular haemorrhage and pseudoaneurysm. Cavitation is a common feature (Figure 8B), but lymphadenopathy is observed in fewer than 20% of patients [65-68]. 

*Cryptococcus neoformans* is a yeast found in avian faeces and soil. There are two varieties: var. *neoformans* and var. *gattii*. The former variety is present worldwide, whilst the latter is mostly seen in the tropics and subtropics, although outbreaks in temperate climates are known [69]. Infections with var. *neoformans* are more common in immunocompromised patients, whilst var. *gattii* is seen in immunocompetent hosts [70].

Diagnosis of pulmonary cryptococcosis is challenging due to a lack of specific distinguishing features on imaging. The most common findings in var. *neoformans* are pulmonary nodules with a peripheral distribution or areas of segmental/lobar consolidation. Miliary disease, cavitation, pleural effusions, and lymph node enlargement are recognised features, more often seen in immunocompromised patients. In immunocompetent patients, the infection usually heals spontaneously [70, 71]. Infections of var. *gattii* more commonly result in focal pulmonary infections (cryptococcomas), although diffuse and bilateral manifestations are known [72]. Example imaging findings are shown in Figure 8D. Infections with var. *neoformans* have been associated with higher mortality than var. *gattii*, although neurological sequelae are more evident in var. *gattii*, reflecting its common cerebral involvement [73].

Advances in highly active antiretroviral therapy and routine prophylaxis have significantly reduced the incidence of *Pneumocystis jirovecii* pneumonia (PCP). Advances in PCP among HIV-infected patients worldwide. However, PCP remains a concern due to its increasing incidence in patients receiving corticosteroids, biological agents, or immunosuppressants [74].

The principal imaging finding is extensive GGO in a central distribution with relative peripheral sparing, although upper lobe predominance is also seen (see Figure 8C, 8F). The extent of GGO is often greater in patients without HIV infection [75, 76].

With more advanced disease, crazy paving and consolidation may develop. Variably sized and shaped pulmonary cysts are evident in up to a third of patients and are associated with spontaneous pneumothoraces. The cysts may resolve after treatment and clearing of infection. Small nodules, tree-in-bud opacities and pleural effusions are uncommon and usually indicate co-infection with another organism. Post recovery, patients may develop interstitial fibrosis [77, 78].

Pulmonary histoplasmosis is caused by the dimorphic fungus *Histoplasma capsulatum*. Pulmonary infection results from inhalation of spores, which germinate, releasing yeasts that proliferate in phagocytosing macrophages, leading to bronchopneumonia and granulomatous inflammation, which later calcify following a specific cellular immune response. Immunocompetent patients usually recover without requiring antifungal treatment, whereas immunocompromised hosts may progress to systemic histoplasmosis and its associated high mortality. Acute infections may not manifest radiologically, but can appear as consolidation and solitary or multiple nodules; disseminated disease is more diffuse micronodular or alveolar consolidation. Chronic disease shows calcified granulomata and lymph nodes, as well as histoplasmomas, which are well-defined nodules with pathognomonic target-like central calcification. It may also progress in patients with concomitant emphysema to upper lobe cavitation and volume loss [79].

*Blastomyces dermatitidis* is endemic to North America and usually causes patchy, ill-defined consolidation in blastomycosis. Immunocompromised patients may develop fulminant disease, with diffuse air-space opacification. Blastomycomas can commonly form as well-defined pulmonary masses. Unlike histoplasmosis, parenchymal and lymph node calcification is uncommon [80].

Pulmonary coccidioidomycosis is caused by *Coccidioides *spp., which are endemic to North, Central, and South America, and most commonly involves the lungs. Imaging features include consolidation (which may be multifocal), nodules, lymph node enlargement, and pleural effusions with interlobular septal thickening on CT. Markedly immunocompromised patients can progress to ARDS with fungaemia, but most other infections will resolve in six weeks. If the infection fails to fully resolve, a chronic mass-like coccidioidoma can form, which can require fine-needle aspiration or biopsy as the differential includes pulmonary malignancy, solitary metastasis, or another granulomatous infection [81].

Complications of Fungal Infection

The host's immune response to aspergillosis, mucormycosis, cryptococcosis, and especially histoplasmosis can cause proliferation of dense fibrous tissue in the mediastinum, causing fibrosing mediastinitis. Typically, a solid soft-tissue density infiltrative lesion with calcifications and variable enhancement is seen in the middle mediastinum (Figure 8E). Cryptococcosis and histoplasmosis can cause disseminated lymphadenopathy and necrosis in immunocompromised patients [44, 79]. 

Pleural effusions are uncommon in pulmonary fungal diseases. Chest wall involvement may be seen in the form of lytic lesions in ribs and vertebrae [44, 45].

Table 4 summarises the imaging appearances of fungal pulmonary infection.

**Table 4:** Summary of the imaging appearances of fungal pulmonary infection. Sources: References [44-81].

Organism / entity	Typical imaging pattern(s)	Key differentiating clue(s)
Angioinvasive aspergillosis	Multifocal poorly defined peripheral nodules coalescing to masses; halo sign; later air-crescent; consolidation	Neutropenic; halo early, air-crescent in recovery
Airway-invasive aspergillosis	Tree-in-bud, nodular airway thickening, bronchopneumonia pattern	Airway-centred
Chronic pulmonary aspergillosis	Upper-lobe cavities ± fungal ball, consolidation, fibrosis, traction bronchiectasis, pleural fibrosis	Pre-existing lung disease (TB, sarcoid, emphysema)
Aspergilloma	Intracavitary soft-tissue mass with air-crescent sign; no change over time	Immunocompetent; underlying TB/sarcoid cavity
Allergic bronchopulmonary aspergillosis (ABPA)	Central bronchiectasis, mucus plugging, 'gloved-finger'; high-attenuation mucus	Asthma or cystic fibrosis
*Candida* spp.	Patchy bilateral nodules with halo, ground-glass opacities (GGO), consolidation, apical sparing; tree-in-bud	Usually colonisation; confident diagnosis needs histology/sterile-site culture
Mucormycosis	Reversed halo sign; consolidation, nodules ± halo, bronchial plugging, cavitation; vascular complications	Reversed halo + rapid progression; lymphadenopathy in <20%
*Cryptococcus* (neoformans/gattii)	Peripheral nodules or segmental/lobar consolidation; cavitation, miliary, effusion, lymphadenopathy if immunocompromised; cryptococcomas (var. gattii)	Exposure to avian droppings/soil
*Pneumocystis jirovecii* pneumonia (PCP)	Central GGO with peripheral sparing; crazy paving/consolidation when advanced; cysts (up to 1/3)	HIV/immunosuppressed; nodules or effusion suggest co-infection; cysts risk pneumothorax
Histoplasmosis	Consolidation, solitary/multiple nodules; chronic: calcified granulomata/nodes, histoplasmomas	Target-like central calcification of histoplasmomas
Blastomycosis	Patchy ill-defined consolidation; masses (blastomycomas)	North America; calcification uncommon
Coccidioidomycosis	Consolidation (± multifocal), nodules, lymphadenopathy, effusion, septal thickening; coccidioidoma	Endemic to North, Central and South America

## Conclusions

Radiological imaging plays a key role in the diagnosis and management of pulmonary infections. A chest X-ray is often the first-line imaging investigation in patients with suspected lung infections, but CT imaging is increasingly becoming indispensable in patients with severe or diagnostically complex infections.

While empirical treatment is often initiated based on clinical likelihood, the time required for microbiology samples to be acquired and analysed means that clinicians often rely on imaging findings to direct more targeted therapy, especially in medical emergencies and immunocompromised patients. Therefore, a better understanding of the imaging features in various types of pulmonary infections is crucial for timely diagnosis and optimum management of patients.
